# Advances in Detection Methods for A‐to‐I RNA Editing

**DOI:** 10.1002/wrna.70014

**Published:** 2025-04-14

**Authors:** Yuxi Yang, Masayuki Sakurai

**Affiliations:** ^1^ Research Institute for Biomedical Sciences Tokyo University of Science Chiba Japan

**Keywords:** A‐to‐I RNA editing, enzyme‐assisted detection, inosine chemical labeling, inosine detection

## Abstract

Adenosine‐to‐inosine (A‐to‐I) RNA editing is a key post‐transcriptional modification that influences gene expression and various cellular processes. Advances in sequencing technologies have greatly contributed to the identification of A‐to‐I editing sites, providing insights into their distribution across coding and non‐coding regions. These developments have facilitated the discovery of functionally relevant editing events and have advanced the understanding of their biological roles. This review presents the evolution of methodologies for RNA editing detection and examines recent advances, including chemically‐assisted, enzyme‐assisted, and quantitative approaches. By evaluating these techniques, we aim to help researchers select the most effective tools for investigating RNA editing and its broader implications in health and disease.

## Graphical abstract

Caption: Early identification of A‐to‐I RNA editing sites was achieved through Sanger sequencing, with the introduction of next‐generation sequencing (NGS) enabling transcriptome‐wide analyses. In recent years, detection approaches have expanded beyond sequencing‐based strategies to include chemically assisted and enzyme‐assisted methods, which offer enhanced specificity and sensitivity for RNA editing analysis.

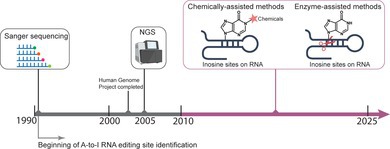

## 1 Introduction

**FIGURE 1:**
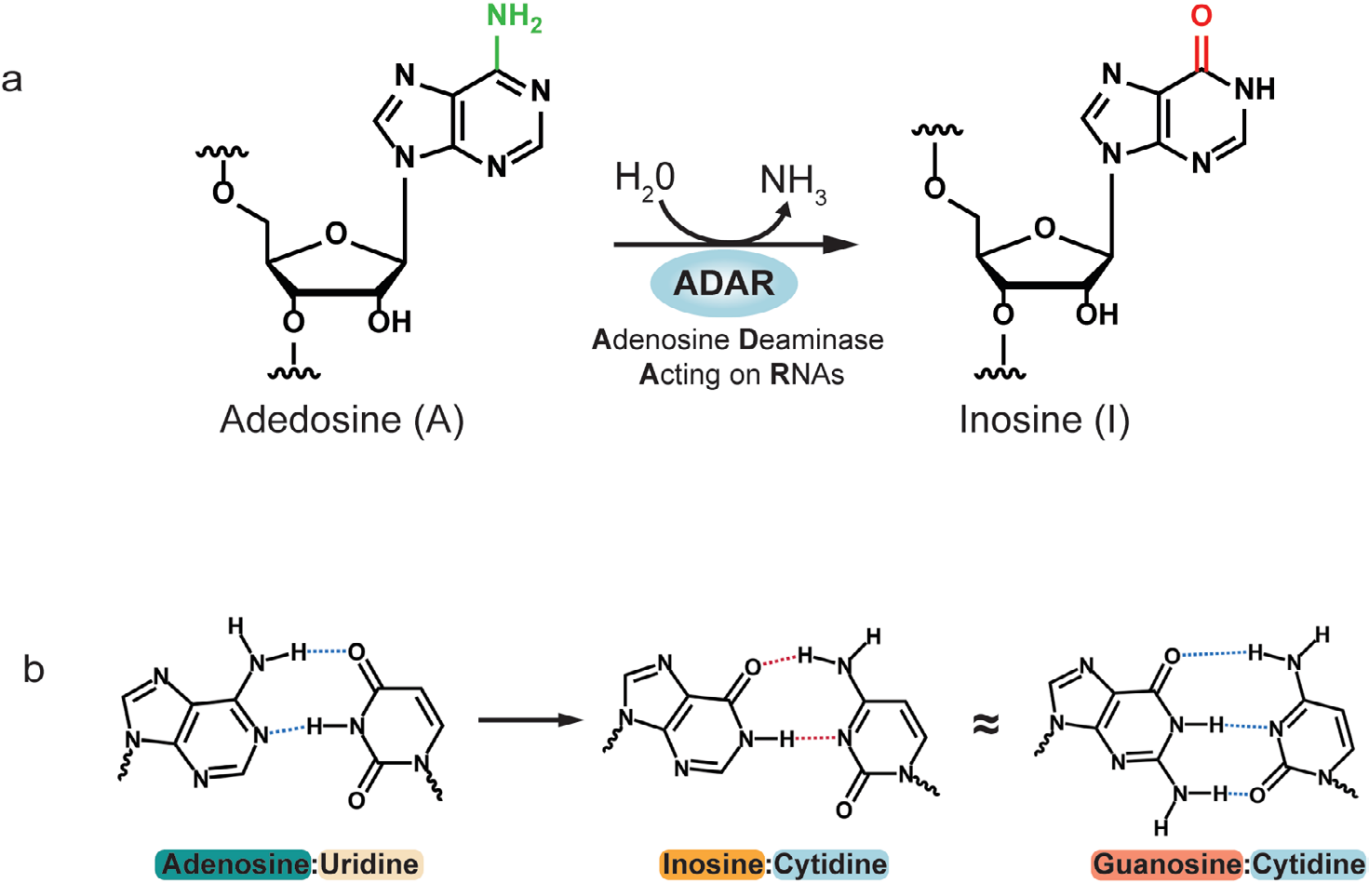
Hydrolytic deamination reaction catalyzed by adenosine deaminase acting on RNAs (ADAR). (a) ADARs catalyze A‐to‐I editing. (b) Inosine pairs with cytidine in a Watson‐Crick‐like configuration, mimicking guanosine–cytidine pairing and disrupting the original adenosine–uridine base pair.

Like DNA and protein, RNA can be modified by various enzymes, which has led to the emergence of epitranscriptomics, the study of RNA modifications and their involvement in the regulation of gene expression (Roundtree and He 2016; Saletore et al. 2012). More than 170 known RNA modifications have been identified (Zhang et al. 2022), influencing RNA processes such as stability, decay, intracellular localization, and translation in a cell type‐ and tissue‐specific manner (Boo and Kim 2020; Schaefer et al. 2017; Vissers et al. 2020). RNA editing is a ubiquitous and crucial post‐transcriptional modification that alters the chemical structure of the base and ribose, and can alter the base‐pairing pattern, resulting in changes in genetic information, RNA structure, and functions, through which cell fate is regulated. There are two types of RNA editing: adenosine to inosine (A‐to‐I) and cytidine to uridine (C‐to‐U) (Christofi and Zaravinos 2019). While C‐to‐U conversion is commonly observed in plants (Knoop 2022), A‐to‐I conversion, catalyzed by adenosine deaminases acting on RNA (ADAR), is predominantly found in animals (Gagnidze et al. 2018) (Figure 1a). ADAR‐mediated A‐to‐I RNA editing is dynamically regulated by various mechanisms to ensure cellular and organismic homeostasis (Vesely and Jantsch 2021). The first A‐to‐I RNA editing sites identified were located in protein‐coding regions of mRNAs (Sommer et al. 1991). In addition to the discovery of editing sites in protein‐coding sequences, widespread editing sites have also been found in non‐coding RNAs such as microRNAs (miRNAs) (Kawahara et al. 2008; Yang et al. 2006) and Alu repetitive elements, belonging to the short interspersed element (SINE) family, located in introns and untranslated regions (UTRs) (Athanasiadis et al. 2004; Bazak et al. 2014; Sakurai et al. 2010). Abnormalities in coding and non‐coding editing sites have been linked to brain development, viral infections, and human diseases (Erdmann et al. 2020; Yang et al. 2021).

A‐to‐I RNA editing is one of the few epitranscriptomic marks that can be easily detected in RNA sequencing (RNA‐seq) data, unlike other RNA modifications. Because inosine has a chemical structure similar to that of guanosine (G), inosine in RNA transcripts acts as G and pairs with C during mRNA splicing and translational processes (Figure 1b). Reverse transcription during RNA‐seq library preparation converts I to G in first‐strand complementary DNA (cDNA). Consequently, when sequencing reads are aligned to the reference genome, A > G changes can be recognized as potential RNA editing sites. With the development of next‐generation sequencing (NGS) technologies and bioinformatics, systematic exploration of new editing sites has been pivotal in the A‐to‐I RNA editing research field (Li et al. 2011). Detection of I modifications offers valuable tools for functional studies. In this review, we summarize the evolution of sequencing technologies for detecting A‐to‐I RNA editing sites and the current advancements in novel detection techniques. We discuss the specific conditions under which each method is most applicable, as well as their respective advantages and limitations. By evaluating these factors, we aim to provide a comprehensive overview of the current landscape of A‐to‐I editing site detection and offer insights into how these methods can be further optimized and applied in different research contexts.

## 2 Advancing Sequencing Technologies and the Discovery of Functional A‐to‐I Editing Sites

The first A‐to‐I RNA editing was detected using Sanger sequencing (Sanger et al. 1977) by comparing nucleotide differences between genomic DNA and cDNA sequences. This approach led to the identification of A‐to‐I editing in the GluA2 subunit of the AMPA glutamate receptor in mice (Sommer et al. 1991), where editing substitutes a glutamine (Q) codon with an arginine (R) codon, altering ion channel permeability (Raymond et al. 1999) (Table 1). Defective Q/R editing results in postnatal death in mice (Brusa et al. 1995), and this lethal phenotype was rescued in mice with a point mutation that changes a glutamate codon to an arginine codon at the Q/R site (Higuchi et al. 2000). Following the discovery of A‐to‐I editing in GluA2, five RNA editing sites were identified in serotonin 5‐hydroxytryptamine_2C_ receptor (5‐HT_2C_R) transcripts (Burns et al. 1997) (Table 1). Editing of 5‐HT_2C_R isoforms resulted in reduced G protein coupling efficiency and agonist binding (Burns et al. 1997; Niswender et al. 1999; Price et al. 2001). In addition, the fully edited 5‐HT_2C_R was highly expressed on the cell surface (Marion et al. 2004).

**TABLE 1:** A‐to‐I RNA editing sites in coding regions with experimentally validated functional consequences. Each site listed has been shown to affect protein structure, cellular signaling, or disease‐related processes.

Gene	Description	Editing site	Editing functions	Refs
*GRIA2*	GluA2 subunit of AMPA glutamate receptor	Q/R	– Reduced Ca^2+^ permeability – Endoplasmic reticulum (ER)	Greger et al. (2002), Raymond et al. (1999), Sommer et al. (1991)
		R/G	– Desensitization restores faster – Reduced splicing efficiency	Lomeli et al. (1994), Schoft et al. (2007)
*GRIA3*	GluA3 subunit of AMPA glutamate receptor	R/G	– Desensitization restores faster	Lomeli et al. (1994)
*GRIA4*	GluA4 subunit of AMPA glutamate receptor	R/G	– Desensitization restores faster	Lomeli et al. (1994)
*GRIK1*	GluK1 subunit of Kainate‐type glutamate receptor	Q/R	– Reduced Ca^2+^ permeability – Effect of fatty acids	Barbon and Barlati (2011), Egebjerg and Heinemann (1993), Wilding (2005)
*GRIK2*	GluK2 subunit of Kainate‐type glutamate receptor	Q/R, I/V, Y/C	– Reduced Ca^2+^ permeability	Burnashev et al. (1995), Köhler et al. (1993)
*HTR2C*	5‐hydroxytryptamine (serotonin) receptor 2C	A, B, C, D, E	– Reduced G‐protein coupling efficiency – Cell surface expression	Burns et al. (1997), Niswender et al. (1999), Price et al. (2001)
*KCNA1*	Voltage‐gated delayed potassium channels (Kv1.1)	I/V	– Recovery of inactivation – Reduced drug and fatty acids blocking	Bhalla et al. (2004), Decher et al. (2010)
*CACNA1D*	Voltage‐dependent calcium channels (Cav1.3)	I/M, Q/R, Y/C	– Reduced Ca^2+^‐feedback	Huang et al. (2012)
*GABRA3*	GABAA receptor subunit α3 (GABRA3)	I/M	– Decreased cell surface levels – Inhibiting the invasion and metastasis of breast cancer cells	Daniel et al. (2011), Gumireddy et al. (2016)
*CADPS1*	Calcium‐dependent secretory activator protein 1 (CAPS1)	E/G	– Increased dense‐core vesicles (DCV)	Li et al. (2009), Miyake et al. (2016)
*NOVA1*	Neuron‐specific RNA binding protein	S/G	– Increased NOVA1 protein stability	Irimia et al. (2014), Sakurai et al. (2014)
*AZIN1*	Antienzyme inhibitor 1	S/G	– Promoting HCC cell proliferation	Chen et al. (2013)
*NEIL1*	DNA repair enzyme NEI‐like protein 1	K/R	– DNA repair	Yeo et al. (2010)
*GLI1*	Glioma‐associated oncogene 1	R/G	– Changed transcriptional activity	Shimokawa et al. (2013)
*CDK13*	Cyclin‐dependent kinase 13 (CDK13)	Q/R	– Promoting HCC occurrence – Promoting thyroid cancer	Dong et al. (2018), Maas et al. (2011), Ramírez‐Moya et al. (2021), Sakurai et al. (2014)
*FLNA*	Filamin A	Q/R	– Regulating vascular contraction and diastolic blood pressure – Regulating cell adhesion, migration, and mechanical properties	Jain et al., (2018), (2022), Levanon et al. (2005)
*FLNB*	Filamin B	M/V	– HCC progression	Chan et al. (2014), Li et al. (2009)
*COPA*	Coatomer subunit alpha	I/V	– Proliferation, migration, and invasion of tumor	Chan et al. (2014), Maas et al. (2011), Peng et al. (2018)
*RHOQ*	Ras homolog family member Q	N/S	– Promoting invasion potential in colorectal cancer	Han et al. (2014)
*BLCAP*	Bladder cancer‐associated protein	Y/C, Q/R, K/R	– Promoting HCC – Facilitating cervical cancer initiation	Chen et al. (2017), Hu et al. (2015), Levanon et al. (2005)
*IGFBP7*	Insulin‐like growth factor‐binding protein 7	K/R, R/G	– Change in sensitivity to proteolysis	Levanon et al. (2005), Sie et al. (2012)
*AR*	Androgen receptor	T/A	– Prostate Cancer progression	Martinez et al. (2008)
*PODXL*	Podocalyxin like	H/R	– Neutralizes the tumorigenic ability of the unedited PODXL in Gastric Cancer	Chan et al. (2016)
*CCNI*	Cyclin I	R/G	– Regard to peptide presentation – T‐cell recognition	Li et al. (2009), Zhang et al. (2018)
*COG3*	Component of oligomeric Golgi complex 3	I/V	– Cell viability – Drug sensitivity	Han et al. (2015), Shah et al. (2009)
*SLC22A3*	Solute carrier family 22 member 3	N/D	– Early development and progression of familial esophageal cancer	Fu et al. (2017), Shah et al. (2009)
*TMEM63B*	Transmembrane protein 63B	Q/R	– Regulating Ca^2+^ permeability and osmosensitivity of channel proteins	Wu et al. (2021)

Although Sanger sequencing is accurate, the discovery of editing sites was slow due to technical limitations and a cumbersome screening process. In the early 2000s, the success of the Human Genome Project, along with the completion of genome sequencing for several model organisms (Adams et al. 2000; Chinwalla et al. 2002), enabled the use of comparative genomics to identify functional genomic elements. The first study using comparative genomics to search for new A‐to‐I RNA editing sites identified 16 editing sites in *Drosophila* (Hoopengardner et al. 2003). Among them, *Shaker* potassium channel genes in *Drosophila* were also found to undergo editing in their human ortholog, *KCNA1* (Kv1.1). Functional studies later demonstrated that the editing converts the isoleucine codon (I) to valine codon (V), accelerating Kv1.1 channel recovery from inactivation and reducing drug and unsaturated fatty acid blocking (Bhalla et al. 2004; Decher et al. 2010) (Table 1). Another study employed comparative genomics and expressed sequence analysis to identify A‐to‐I editing sites in four human genes: FLNA, BLCAP, CYFIP2, and IGFBP7 (Levanon et al. 2005) (Table 1 and Table 2). Subsequent research gradually revealed the functional significance of these editing sites. Filamin A (FLNA) is an actin crosslinking protein that undergoes A‐to‐I RNA editing at exon 42, resulting in a Q to R amino acid change in its 22nd immunoglobulin (Ig)‐like domain (Levanon et al. 2005; Stossel et al. 2001; Zhou et al. 2010). This modification has been implicated in vascular contraction and diastolic blood pressure regulation (Jain et al. 2018) was later shown to influence cell adhesion, migration, and mechanical properties (Jain et al. 2022). The Bladder cancer‐associated protein (BLCAP) gene undergoes A‐to‐I RNA editing, which has been shown to contribute to hepatocarcinogenesis (Chen et al. 2017; Hu et al. 2015). The A‐to‐I editing site in cytoplasmic FMRP interacting protein 2 (CYFIP2) has been implicated in aging (Nicholas et al. 2010). However, the biological significance of CYFIP2 editing remains unclear. Editing of insulin‐like growth factor‐binding protein‐7 (IGFBP7) transcripts has been shown to influence the protein's susceptibility to proteolytic cleavage (Sie et al. 2012). The discovery of these editing sites and the exploration of their functions highlight that RNA editing is not limited to genes encoding ion channel proteins. Furthermore, investigating RNA editing beyond the nervous system is important.

**TABLE 2:** A‐to‐I RNA editing sites identified through sequencing that currently lack functional characterization. These entries are commonly used as references in editing studies but have not been linked to specific biological functions.

Gene	Description	Editing site	Refs	Gene	Description	Editing site	Refs
*CYFIP2*	Cytoplasmic FMR1‐interacting protein 2	K/E	Levanon et al. (2005)	*METTL10*	Methyltransferase like 10	L/L, T/A, G/G	Sakurai et al. (2014)
*SON*	SON DNA‐binding protein	T/A, L/L, P/P, R/G A/A	Peng et al. (2012); Sakurai et al. (2014)	*GPT2*	Glutamic–pyruvic transaminase 2	R/G, K/R, K/E, S/S, S/G	Sakurai et al. (2014)
*UNC80*	Protein unc‐80 homolog	S/G	Gabay et al. (2022)	*BEST1*	Bestrophin‐1	I/V	Sakurai et al. (2014)
*SPEG*	SPEG complex locus	S/G	Gabay et al. (2022)	*CPT1A*	Carnitine palmitoyltransferase 1A	E/G	Sakurai et al. (2014)
*UBE2O*	Ubiquitin conjugating enzyme E2 O	S/G	Cruz et al. (2020)	*FAM90A1*	Family with sequence similarity 90 member A1	E/G	Sakurai et al. (2014)
*DACT3*	Dapper homolog 3	R/G	Cruz et al. (2020)	*PUS1*	Pseudouridine synthase 1	A/A, N/D, S/S	Sakurai et al. (2014)
*ALPL*	Alkaline phosphatase	P/P	Li et al. (2009)	*TPTE2P1*	Transmembrane phosphoinositide 3‐phosphatase and tensin homolog 2 pseudogene 1	V/V	Sakurai et al. (2014)
*C1orf175*	Chromosome 1 open reading frame 175	K/R	Li et al. (2009)	*SLC38A6*	Solute carrier family 38 member 6	L/L	Sakurai et al. (2014)
*HMCN1*	Hemicentin‐1	K/E	Li et al. (2009)	*ANKDD1A*	Ankyrin repeat and death domain containing 1A	Q/R	Sakurai et al. (2014)
*ZNF638*	Zinc finger protein 638	K/K	Li et al. (2009)	*PDCD7*	Programmed cell death protein 7	A/A, Q/R	Sakurai et al. (2014); Wen et al. (2021)
*BIN1*	Bridging integrator 1	K/R	Li et al. (2009)	*GIPC1*	GIPC PDZ domain‐containing family member 1	T/A, P/P	Sakurai et al. (2014)
*COL6A3*	Collagen, type VI, alpha 3	P/P	Li et al. (2009)	*MPST*	Mercaptopyruvate sulfurtransferase	V/V, S/S	Sakurai et al. (2014)
*MSL3P1*	MSL3 pseudogene 1	K/K, V/V	Peng et al. (2012)	*IRS4*	Insulin receptor substrate 4	Y/C	Gabay et al. (2022)
*TTLL3*	Tubulin tyrosine ligase like 3	K/R	Li et al. (2009)	*CACNG8*	Calcium voltage‐gated channel auxiliary subunit gamma 8	S/G	Wen et al. (2021)
*ATXN7*	Ataxin‐7	K/R	Li et al. (2009)	*NUP214*	Nucleoporin 214	S/G	Gabay et al. (2022)
*NUDT16*	Nudix hydrolase 16	V/V	Li et al. (2009)	*CNNM1*	Cyclin and CBS domain divalent metal cation transport mediator 1	S/G	Gabay et al. (2022)
*LIPH*	Lipase member H	K/K	Li et al. (2009)	*SEMA5B*	Semaphorin 5B	Stop/W	Gabay et al. (2022)
*SH3TC1*	SH3 domain and tetratricopeptide repeat‐containing protein 1	K/R	Li et al. (2009)	*ASNS*	Asparagine synthetase (glutamine‐hydrolyzing)	Y/C	Gabay et al. (2022)
*KIAA0947*	KIAA0947 gene product	Q/Q	Li et al. (2009)	*SRP9*	Signal recognition particle 9	I/M	Shah et al. (2009)
*NIPBL*	NIPBL cohesin loading factor	V/V	Li et al. (2009)	*PDLIM*	PDZ and LIM domain proteins	N/S	Lee et al. (2017)
*CUX1*	Homeobox protein cut‐like 1	P/P	Li et al. (2009)	*APMAP*	Adipocyte plasma membrane associated protein	I/V	Lee et al. (2017)
*ATP6V0E2*	ATPase H+ transporting V0 subunit e2	K/E, R/R, I/M, R/G, H/R	Sakurai et al. (2010)	*ZNF358*	Zinc finger protein 358	K/R	Lee et al. (2017)
*OTUD6B*	OTU deubiquitinase 6B	Q/Q	Li et al. (2009)	*CABP1*	Calcium binding protein 1	Q/R	Wen et al. (2021)
*PTK2*	Protein tyrosine kinase 2	T/A	Li et al. (2009)	*METTL10*	Methyltransferase like 10	T/A	Wen et al. (2021)
*FBXL6*	F‐box/LRR‐repeat protein 6	Stop/W	Li et al. (2009)	*LRP4*	LDL receptor related protein 4	S/G	Wen et al. (2021)
*ZNF169*	Zinc finger protein 169	Q/Q, H/R	Levanon et al. (2004); Sakurai et al. (2010)	*ACCN2*	Acid sensing ion channel subunit 1	T/A	Wen et al. (2021)
*CRB2*	Crumbs cell polarity complex component 2	T/A	Li et al. (2009)	*C1orf86*	FA core complex associated protein 20	A/A	Kang et al. (2015)
*RSU1*	Ras suppressor protein 1	M/V	Li et al. (2009)	*IL6R*	Interleukin 6 receptor	A/A	Kang et al. (2015)
*KCNMA1*	Potassium calcium‐activated channel subfamily M alpha 1	S/G	Li et al. (2009)	*ZBTB7B*	Zinc finger and BTB domain containing 7B	Stop/Stop	Kang et al. (2015)
*KIF20B*	Kinesin family member 20B	K/R	Li et al. (2009)	*HSD17B7*	Hydroxysteroid 17‐beta dehydrogenase 7	L/L	Kang et al. (2015)
*TCP11L1*	T‐complex protein 11‐like protein 1	L/L	Li et al. (2009)	*CFH*	Complement factor H	Y/C	Kang et al. (2015)
*ARFGAP2*	ADP‐ribosylation factor GTPase‐activating protein 2	Q/R	Li et al. (2009)	*TACC2*	Transforming acidic coiled‐coil containing protein 2	T/T	Kang et al. (2015)
*CD6*	T‐cell differentiation antigen CD6	S/G	Li et al. (2009)	*TUBGCP2*	Tubulin gamma complex associated protein 2	N/S, R/G	Kang et al. (2015)
*GANAB*	Neutral alpha‐glucosidase AB	Q/R	Li et al. (2009)	*C11orf80*	Chromosome 11 open reading frame 80	S/G, S/S	Kang et al. (2015)
*BSCL2*	BSCL2 lipid droplet biogenesis associated, seipin	E/E	Li et al. (2009)	*ALG8*	ALG8 alpha‐1,3‐glucosyltransferase	Q/Q	Kang et al. (2015)
*NUMA1*	Nuclear mitotic apparatus protein 1	T/A	Li et al. (2009)	*NOP2*	NOP2 nucleolar protein	Q/R	Kang et al. (2015)
*PLEKHA9*	Putative protein PLEKHA9	E/E, E/G, P/P, R/G, H/R	Peng et al. (2012)	*ACRBP*	Acrosin binding protein	T/A	Kang et al. (2015)
*OS9*	Protein OS‐9	E/G	Li et al. (2009)	*SLC39A5*	Solute carrier family 39 member 5	S/S	Kang et al. (2015)
*KIAA1033*	KIAA1033 gene product	T/A	Li et al. (2009)	*SLC35E3*	Solute carrier family 35 member E3	I/M	Kang et al. (2015)
*UTP14C*	U3 small nucleolar RNA‐associated protein 14 homolog C	S/G, Q/R	Peng et al. (2012)	*CHFR*	Checkpoint with forkhead and ring finger domains	S/G	Kang et al. (2015)
*PML*	Promyelocytic leukemia protein	A/A	Li et al. (2009)	*RAD51B*	RAD51 paralog B	L/L	Kang et al. (2015)
*MEX3B*	RNA‐binding protein MEX3B	Q/R	Li et al. (2009)	*CTDNEP1*	CTD nuclear envelope phosphatase 1	N/D	Kang et al. (2015)
*SPSB3*	SPRY domain‐containing SOCS box protein 3, SSB‐3	K/R	Li et al. (2009)	*SLFN12L*	Schlafen family member 12 like	Stop/W	Kang et al. (2015)
*ZNF587B*	Zinc finger protein 587B	I/M, K/E, K/R, Stop/Stop	Kang et al. (2015)	*NARF*	Nuclear prelamin A recognition factor	S/G, Stop/W, T/A, V/V, I/V, S/S, Q/R	Kang et al. (2015)
*C1QL1*	Complement C1q‐like protein 1	T/A, Q/R	Sie and Maas (2009)	*TBXA2R*	Thromboxane A2 receptor	A/A, S/S	Kang et al. (2015)
*CARM1*	Histone‐arginine methyltransferase CARM1	R/R	Li et al. (2009)	*IL12RB1*	Interleukin 12 receptor subunit beta 1	R/G, P/P, S/S I/V	Kang et al. (2015)
*STRN4*	Striatin‐4	R/G	Li et al. (2009)	*ZNF714*	Zinc finger protein 714	N/D, N/S, Q/Q	Kang et al. (2015)
*TMEM230*	Transmembrane protein 230	S/G, H/R, L/L	Levanon et al. (2004)	*ZNF429*	Zinc finger protein 429	K/E, N/S	Kang et al. (2015)
*SS18L1*	Calcium‐responsive transactivator	S/G	Li et al. (2009)	*ZNF91*	Zinc finger protein 91	T/T	Kang et al. (2015)
*ZNF70*	Zinc finger protein 70	Y/C	Li et al. (2009)	*MAP4K1*	Mitogen‐activated protein kinase 1	S/S	Kang et al. (2015)
*UBA1*	Ubiquitin‐like modifier‐activating enzyme 1	L/L	Kim et al. (2004); Levanon et al. (2004)	*GPT2*	Alanine aminotransferase 2, ALT2	K/K	Li et al. (2009)
*TRO*	Trophinin	S/G	Li et al. (2009)	*TGOLN2*	Trans‐golgi network protein 2	D/G, K/K	Kang et al. (2015)
*PLCH2*	1‐phosphatidylinositol 4,5‐bisphosphate phosphodiesterase eta‐2	R/G	Sakurai et al. (2014)	*SULT1C2*	Sulfotransferase family 1C member 2	T/T, S/G, K/E, K/R	Kang et al. (2015)
*NCSTN*	Nicastrin	S/G	Sakurai et al. (2014)	*PID1*	Phosphotyrosine interaction domain containing 1	Q/Q	Kang et al. (2015)
*ZNF669*	Zinc finger protein 669	N/D, Y/C	Sakurai et al. (2014)	*IFNAR2*	Interferon alpha and beta receptor subunit 2	Q/Q, N/S, K/R	Kang et al. (2015)
*ZMYM6*	Zinc finger MYM‐type protein 6	K/R, V/V	Sakurai et al. (2014)	*C22orf39*	Chromosome 22 open reading frame 39	T/A, S/G	Kang et al. (2015)
*PPIL3*	Peptidyl‐prolyl cis‐trans isomerase‐like 3, PPIase	S/G, V/V	Sakurai et al. (2014)	*NIPSNAP1*	Nipsnap homolog 1	K/E	Kang et al. (2015)
*LRRFIP1*	Leucine‐rich repeat flightless‐interacting protein 1, LRR FLII‐interacting protein 1	K/R	Sakurai et al. (2014)	*FAM193A*	Family with sequence similarity 193 member A	H/R	Kang et al. (2015)
*METTL6*	tRNA N(3)‐methylcytidine methyltransferase METTL6	A/A, H/R	Sakurai et al. (2014)	*SEPP1*	Selenoprotein P	R/G, Q/R, N/S, H/R	Kang et al. (2015)
*LMLN*	Leishmanolysin like peptidase	P/P	Sakurai et al. (2014)	*LYRM4*	LYR motif containing 4	K/R	Kang et al. (2015)
*RMND5B*	Required for meiotic nuclear division 5 homolog B	S/G	Sakurai et al. (2014)	*KIF13A*	Kinesin family member 13A	K/R	Kang et al. (2015)
*GRM4*	Metabotropic glutamate receptor 4, mGluR4	Q/R	Sakurai et al. (2014)	*NPC1L1*	NPC1 like intracellular cholesterol transporter 1	S/G	Kang et al. (2015)
*FLJ44955(uc003qkz.1)*	FLJ44955 protein	K/R	Sakurai et al. (2014)	*NOS3*	Nitric oxide synthase 3	S/G	Kang et al. (2015)
*TNRC18*	Trinucleotide repeat‐containing gene 18 protein	E/G	Sakurai et al. (2014)	*C8orf44‐SGK3;C8orf44*	Chromosome 8 open reading frame 44	N/R	Kang et al. (2015)
*STYXL1*	Serine/threonine/tyrosine‐interacting‐like protein 1	Stop/W	Sakurai et al. (2014)	*PARP10*	Poly(ADP‐ribose) polymerase family member 10	N/R	Kang et al. (2015)
*XKR6*	XK‐related protein 6	R/G	Sakurai et al. (2014)	*CSF2RA*	Colony stimulating factor 2 receptor subunit alpha	H/R	Kang et al. (2015)
*SORBS1*	Sorbin and SH3 domain‐containing protein 1	V/V	Sakurai et al. (2014)	*ZN358*	Zinc finger protein 358	K/R	Solomon et al. (2014)

*Note:* This table may serve as a reference for future studies aiming to investigate the potential functional roles of currently uncharacterized editing sites.

Next, methods for aligning expressed sequence tag (EST) libraries to genome sequences emerged. Four studies aligned millions of human ESTs and full‐length cDNA transcripts to genome sequences and assessed A‐to‐G mismatches (Athanasiadis et al. 2004; Blow et al. 2004; Kim et al. 2004; Levanon et al. 2004). More than 10,000 RNA editing sites have been identified, most concentrated in the Alu sequences of non‐coding regions of genes. The emergence of NGS technology has increased further the efficiency of RNA editing site detection, enabling the de novo identification of editing sites without prior information and leading to an exponential increase in the number of RNA editing sites (Li et al. 2009). As NGS continues to uncover a growing number of A‐to‐I editing sites, many have been functionally characterized, while others remain unexplored. To illustrate this, we have compiled a list of other identified editing sites with significant functional roles (Table 1). Furthermore, we have cataloged additional editing sites with still unknown functions (Table 2). These tables provide a comprehensive overview of both established and yet‐to‐be‐explored A‐to‐I RNA editing sites, underscoring the need for continued research to fully understand their biological implications.

**FIGURE 2:**
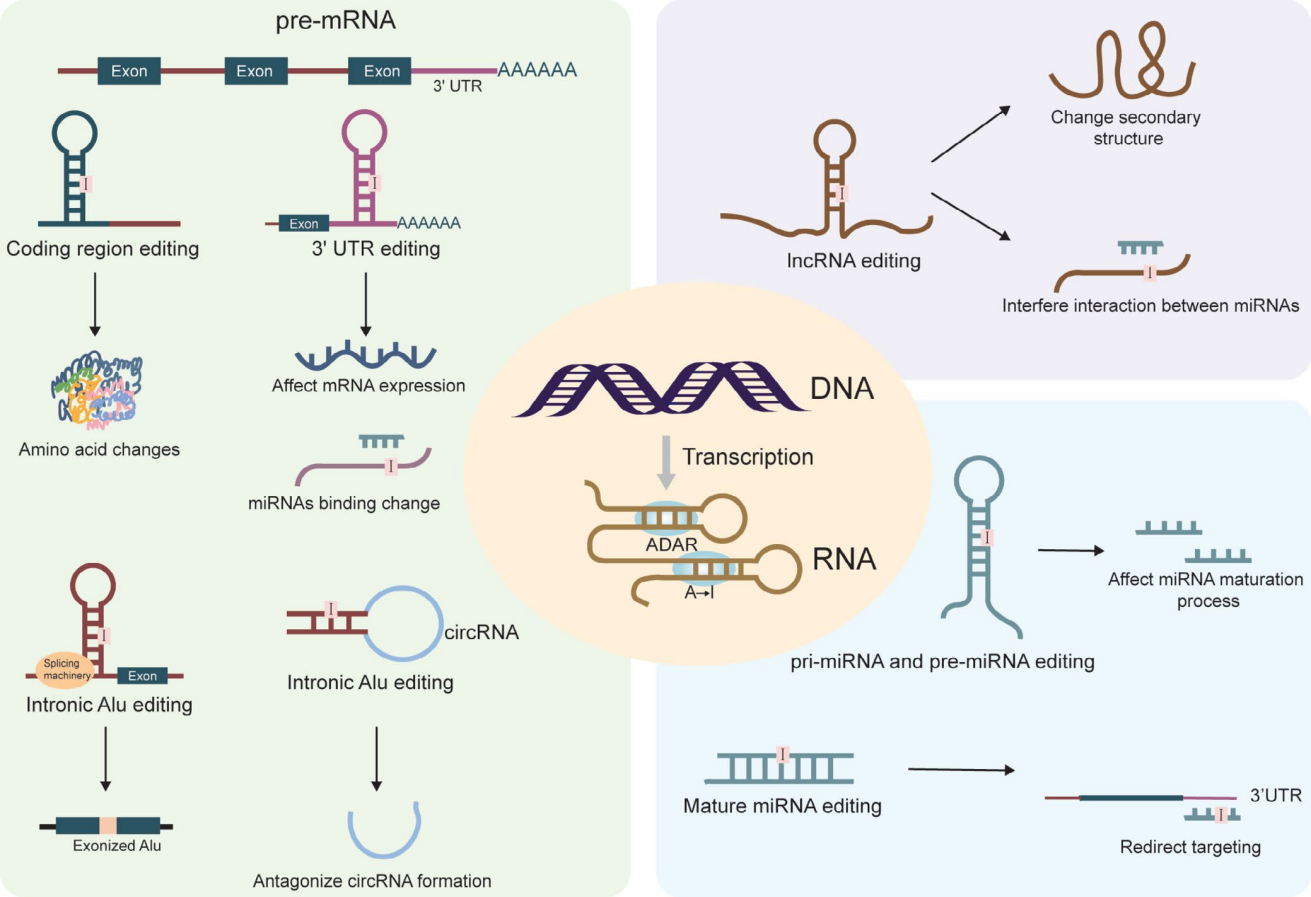
Schematic representation of functional consequences of A‐to‐I RNA editing. Editing in pre‐mRNA (left) can affect coding sequences (amino acid changes), 3′UTRs (mRNA expression or miRNA binding), and intronic Alu elements (exonization or circRNA regulation). Editing in lncRNAs (upper right) may alter secondary structure or RNA–RNA interactions. Editing in miRNAs (lower right) can impact maturation or redirect targeting.

As RNA sequencing became a widely used methodology, emerging findings suggest that RNA editing is not restricted to coding regions but is also widely present in non‐coding regions of the genome (Li et al. 2009), laying the foundation for subsequent studies on A‐to‐I editing in non‐coding RNAs. Following this, research on Alu editing further confirmed that the editing of intronic Alu elements may lead to their exonization (Lev‐Maor et al. 2007; Sakurai et al. 2010) (Figure 2). With the development of NGS, it has become possible to perform more comprehensive transcriptome analyses, leading to the discovery of numerous A‐to‐I editing sites within non‐coding RNA. These findings have prompted further investigations into how RNA editing modulates the function of non‐coding RNAs in various biological processes. For example, RNA editing can inhibit miRNA maturation by blocking the cleavage of primary miRNAs (pri‐miRNAs) and precursor miRNAs (pre‐miRNAs) (Iizasa et al. 2010; Kawahara, Zinshteyn, Chendrimada, et al. 2007; Kawahara et al. 2008; W. Yang et al. 2006). The ability of editing mature miRNAs to redirect their targets is conferred when the editing site is located in the seed region (Kawahara, Zinshteyn, Sethupathy, et al. 2007; Pinto et al. 2017; Vesely et al. 2012; Wang et al. 2017) (Figure 2). A‐to‐I editing of long non‐coding RNAs (lncRNAs) affects their secondary structure (Gong et al. 2017), which can determine their functions in guiding, scaffolding, and isolating regulatory RNAs or proteins (Bhartiya and Scaria 2016). Editing of lncRNAs may generate new miRNA‐targeting sites or remove miRNA‐targeting sites on lncRNAs, thereby regulating mRNA expression or inhibiting miRNA decoy functions (Gong et al. 2017) (Figure 2). Beyond noncoding RNAs, A‐to‐I editing also plays regulatory roles in mRNA UTRs. Editing in the 3′UTR can reduce translation efficiency and downregulate gene expression in human cells (Yang et al. 2017). This modification can also modulate mRNA degradation by stabilizing RNA secondary structure, which in turn alters the accessibility of miRNA‐binding sites (Brümmer et al. 2017) (Figure 2). Furthermore, A‐to‐I RNA editing events also occur in intronic Alu elements bracketing circRNAs (single‐stranded RNAs that form a continuous closed loop). Hyperediting antagonizes circRNA formation by disrupting annealing between introns (Ivanov et al. 2015) (Figure 2).

NGS has become a powerful tool for identifying A‐to‐I RNA editing sites by aligning RNA sequences to the reference genome and detecting A‐to‐G mismatches. However, distinguishing true editing events from false positives remains a major challenge. This issue became particularly evident in 2011, when a study using matched RNA‐DNA sequencing reported over 10,000 exonic sites in which the RNA sequence did not match the DNA sequence (Li et al. 2011). However, subsequent analyses showed that many of these RNA‐DNA differences were false positives, caused by sequencing errors, misalignments, and technical biases rather than genuine A‐to‐I modifications (Kleinman and Majewski 2012; Lin et al. 2012; Pickrell et al. 2012). To improve the accuracy of RNA editing site identification, researchers have developed more refined strategies. While computational methods have been enhanced to filter out sequencing errors, misalignments, and SNP interference (Bahn et al. 2012; Park et al. 2012), these approaches still rely on indirect inference rather than direct detection of inosine. As a result, additional efforts have focused on improving detection techniques themselves, leading to the development of more precise methods that can provide direct evidence of RNA editing and enhance the reliability of NGS‐based analysis. These methods can be categorized into three main types: chemically‐assisted detection methods, enzyme‐assisted detection, and quantitative methods.

## 3 Innovative Detection Methods for A‐to‐I RNA Editing

### 3.1 Chemically‐Assisted Detection Methods

**FIGURE 3:**
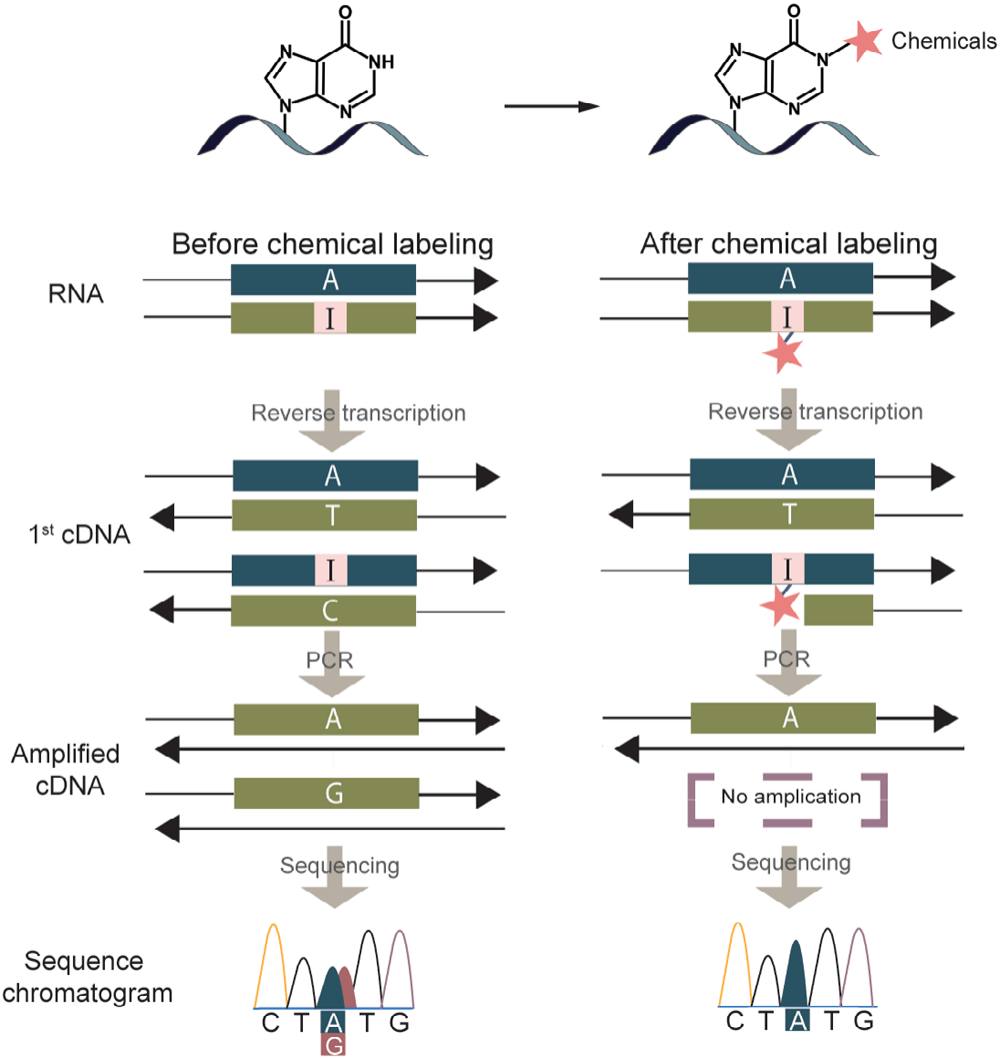
Schematic representation of the principle underlying chemically assisted methods for inosine detection, using chemical labeling and sequencing to distinguish true inosine sites.

Chemical modification techniques that alter inosine base pairing have been widely employed for the detection of inosine. These methods were originally developed to reduce background noise and false positives during sequencing, thereby enabling accurate identification of true inosine editing sites. The principle involves primer extension during reverse transcription (Figure 3). Unlike other RNA modifications, inosine pairs with cytidine (C) during reverse transcription, allowing the synthesis of full‐length cDNA. However, when a compound specifically reacts with inosine, it creates steric hindrance, preventing inosine from pairing with C and resulting in truncated cDNA. Consequently, sequencing reveals a reduction in guanosine (G) signals, which helps distinguish true editing sites.

**FIGURE 4:**
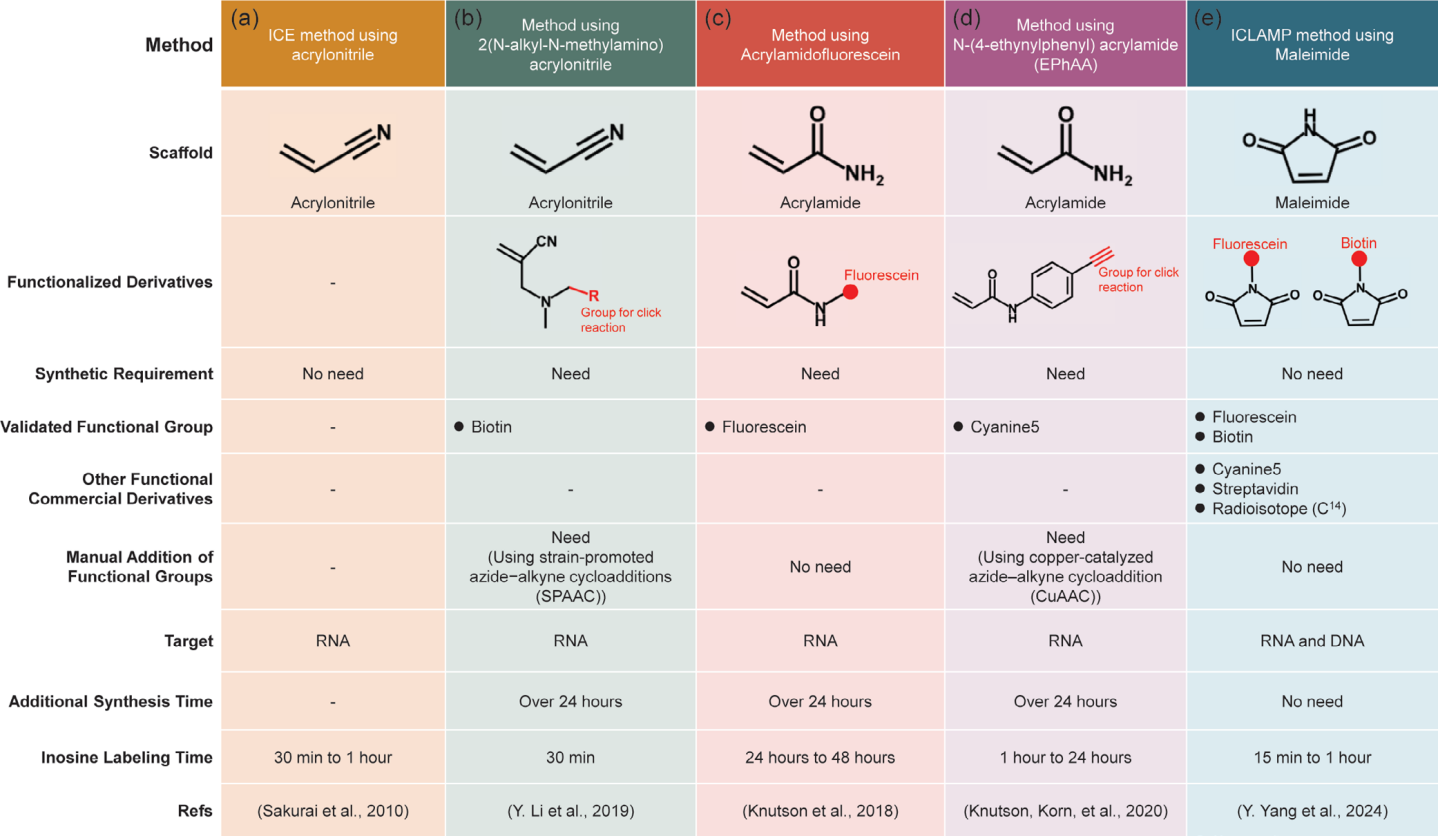
Comparison of various chemically assisted methods for inosine detection using different chemical scaffolds and their functionalized derivatives.

The first method is the inosine chemical erasing (ICE) method (Figure 4a). This method relies on a chemical reaction in which acrylonitrile cyanoethylates inosine to form 1‐cyanoethylinosine via Michael addition, resulting in the inhibition of reverse transcriptase elongation at inosine nucleotides (Sakurai et al. 2010; Yoshida and Ukita 1965). Total RNA treated with acrylonitrile is amplified by PCR and sequenced directly. The A‐to‐I editing sites are predicted by comparing untreated cDNA sequencing chromatograms (A and G mixed signals) with a cyanoethylated cDNA sequencing chromatogram (erased G signals) (Sakurai et al. 2010). The ICE method does not require genomic DNA as a reference. It can distinguish inosine from signals generated by sequencing errors and false positives; however, the limitation of this method is that it can only be applied to sequences of interest. To identify new sites, a new strategy was developed combining ICE with NGS, resulting in the ICE‐seq method, which has detected ~20,000 new editing sites (Sakurai et al. 2014). While the ICE method has been criticized because of its off‐target effects, such as the tendency of acrylonitrile to react non‐specifically with pseudouridine (Ψ), cyanoethylation of Ψ does not inhibit reverse transcriptase elongation (Mengel‐Jørgensen and Kirpekar 2002). Therefore, Ψ is not detected by primer extension, and this does not affect the specificity of ICE in detecting inosine. The true limitation of the ICE method lies in the chemical stability of acrylonitrile, which prevents it from being further modified to link functional groups such as biotin or streptavidin for inosine enrichment. This makes it challenging to use ICE to detect low‐abundance RNAs. Nonetheless, ICE is effective for rapidly detecting specific target sites or confirming editing efficiency.

Building on the ICE method, the Nano ICE‐Seq protocol extends this approach by utilizing the selective reactivity of acrylonitrile with nanopore dRNA‐seq (Ramasamy et al. 2022), which permits a more precise detection of inosine modifications.

Acrylonitrile derivatives were subsequently synthesized to achieve conjugation of functional groups (Y. Li et al. 2019) (Figure 4b). These derivatives, designed with an azidoethyl or propargyl group, enable the attachment of biotin through “click” chemistry, facilitating the enrichment of inosine‐containing RNA. While these derivatives have been tested in mouse brain total RNA using RT‐qPCR to confirm their ability to enrich inosine‐containing RNA, their specificity and potential off‐target effects in complex biological samples remain unexamined. Although these reagents enable selective enrichment, their ability to recover site‐specific inosine‐containing fragments with high fidelity has not been established, limiting their utility in transcriptome‐wide analyses. Moreover, acrylonitrile serves as a promising scaffold for chemical labeling of inosine; synthesis of these reagents requires several synthetic and purification steps.

Acrylamide derivatives such as acrylamidofluorescein and *N*‐(4‐ethynylphenyl)acrylamide (EPhAA) were introduced to enhance functional group labeling (Figure 4c,d). Acrylamidofluorescein enables fluorescent detection and enrichment of inosine in RNA using antifluorescein antibodies, but its reactivity with inosine and labeling efficiency are significantly lower than those of acrylonitrile (Knutson et al. 2018). Like acrylonitrile, it exhibits nonspecific reactivity toward Ψ. Additionally, it has not been tested in complex biological samples, leaving its practicality in such contexts uncertain. Building on the acrylamide scaffold, N‐(4‐ethynylphenyl)acrylamide (EPhAA) was synthesized in a single step and is compatible with “click” chemistry, allowing flexible attachment of fluorescent probes at editing sites (Knutson, Korn, et al. 2020). Although EPhAA offers improved reactivity with inosine, it is still dependent on chemical synthesis, which adds complexity to the process. EPhAA also exhibits nonspecific reactivity toward Ψ. While the reactivity of EPhAA has been validated with synthetic RNA oligonucleotides, it has not been assessed in complex biological samples. Its application remains limited to visualization experiments, and the lack of an enrichment strategy prevents its use in detecting low‐abundance editing events.

Recent advances in inosine detection have led to the development of the inosine chemical labeling and affinity molecular purification (ICLAMP) method (Yang et al. 2024) (Figure 4e), which builds on the principles of chemical labeling while overcoming the limitations of previous approaches. Unlike earlier methods that struggled with the chemical stability of acrylonitrile and the complex synthesis needed for functional group conjugation, ICLAMP utilizes commercially available maleimide derivatives to label inosine with high specificity. The reactivity of maleimide with inosine enables efficient fluorescent labeling and biotin‐tagging of inosine residues in both RNA and DNA. The method can increase the precision and sensitivity of inosine detection, especially the detection of inosine in low‐abundance RNA and DNA. However, its potential nonspecific reactivity with Ψ has not yet been evaluated. The introduction of maleimide reactivity through the ICLAMP method expands chemically assisted approaches beyond the acrylonitrile framework. Given its common use in the chemical modification of proteins, many commercially available maleimide derivatives are widely available and could be used to develop new technologies for the detection of inosine editing sites. For instance, fluorophore‐maleimide could be used for cell staining, allowing visualization of inosine modifications within living cells and enabling real‐time observation of RNA editing events. Additionally, streptavidin‐maleimide derivatives could make ICLAMP applications more flexible, and radiolabeled maleimide derivatives available on the market could be explored for their potential to increase the sensitivity and precision of detection. Although hypothetical and lacking direct experimental evidence, these ideas open promising directions for expanding the versatility of the ICLAMP method and developing innovative applications for the study of A‐to‐I editing.

### 3.2 Enzyme‐Assisted Detection Methods

**FIGURE 5:**
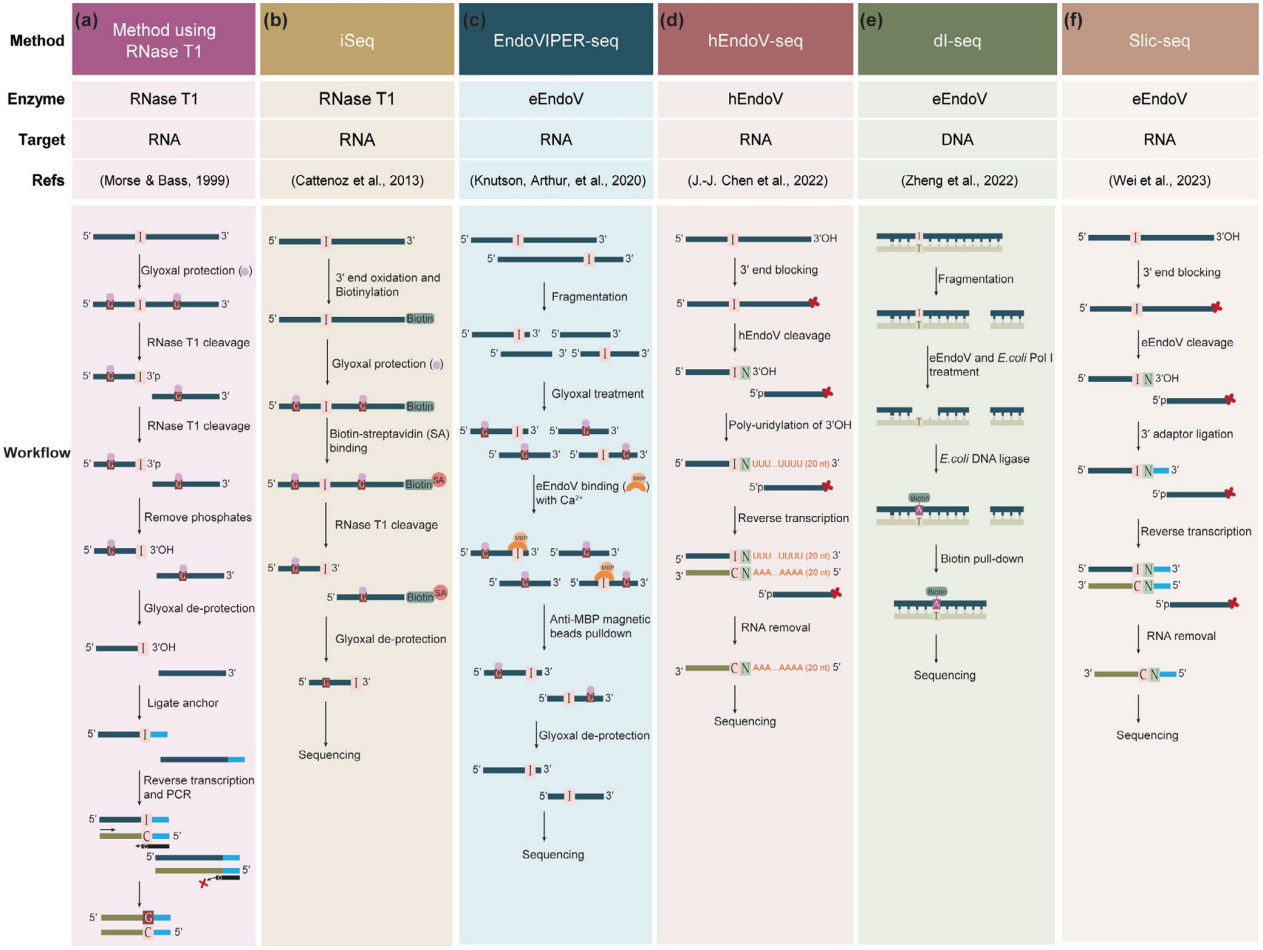
Comparison of enzyme‐assisted inosine detection methods with workflow schematics for each approach.

Enzyme‐assisted methods for detecting inosine take advantage of an enzyme's ability to recognize and cleave specifically inosine‐containing RNA. The first method utilized RNase T1 to cleave inosine‐containing sites. Typically, RNase T1 cleaves guanosine and inosine. Glyoxal/borate was used to protect guanosines from cleavage and form a compound (G*) (Morse 2004; Morse and Bass 1997, 1999) (Figure 5a). Following the removal of the G* adduct from the RNA post‐cleavage, an anchor sequence is ligated to the fragment containing inosine. This modified fragment serves as the template for reverse transcription and subsequent PCR amplification.

Another method using RNase T1, named iSeq, for detecting inosine involves an additional RNA biotinylation step, followed by binding of the modified RNA to magnetic beads (Figure 5b). RNA is similarly treated with glyoxal and borate to protect guanosines, leaving inosines unprotected and susceptible to RNase T1 cleavage (Cattenoz et al. 2013). Cleavage releases fragments with 3′ inosines, which can then be selectively eluted and prepared for further analysis. However, precisely modulating glyoxal/boric acid treatments to make guanosine resistant to RNase T1 remains challenging because guanosines make up ~25% of RNA bases and it is difficult to modify all of them. As a result, incomplete guanosine protection can lead to non‐specific cleavage, limiting the method's accuracy in complex RNA samples.

As an alternative to RNase T1‐based methods, enzyme‐assisted detection of inosine has increasingly relied on Endonuclease V (EndoV). *Escherichia coli* EndoV (eEndoV) cleaves inosine at the 3′ phosphodiester bond in both DNA and RNA, with a preference for DNA. Human EndoV (hEndoV) also cleaves at the 3′ end of inosine but shows a preference for single‐stranded RNA (Morita et al. 2013; Vik et al. 2013). These enzymes enhance the precision of inosine detection, and several methods have been developed based on these cleavage properties.

EndoVIPER‐seq is a method developed to enhance the detection of A‐to‐I RNA editing sites by enriching inosine‐containing transcripts (Knutson, Arthur, et al. 2020) (Figure 5c). This technique takes advantage of commercially available recombinant eEndoV, fused to maltose‐binding protein (MBP). In the presence of Ca^2+^, eEndoV binds to inosine without cleaving the RNA, allowing for selective capture of the inosine‐containing RNA by anti‐MBP magnetic beads. Glyoxal is employed to denature RNA secondary structures, ensuring efficient inosine capture. The enrichment capability of EndoVIPER‐seq makes it well suited for detecting low‐abundance inosine‐containing RNAs. Nevertheless, since the method is based on binding rather than cleavage, it lacks single‐base resolution for pinpointing editing sites.

The hEndoV‐seq method offers precise single‐base resolution detection of A‐to‐I RNA editing sites (Chen et al. 2022) (Figure 5d). This technique involves blocking the 3′‐OH group of RNA, followed by specific cleavage at inosine sites by hEndoV. The resulting 3′‐OH ends are then poly‐uridylated, facilitating sequencing. This approach avoids the complexity and potential degradation associated with chemical treatments, providing a straightforward and efficient method for identifying A‐to‐I editing sites across the transcriptome. Although hEndoV‐seq enables transcriptome‐wide detection with single‐nucleotide resolution, its multi‐step workflow and the cleavage efficiency of hEndoV being affected by RNA secondary structure and local sequence context may contribute to false signals in complex samples.

Another approach, the dI‐seq method, has been developed to identify deoxyinosine (dI) modification sites in DNA (Zheng et al. 2022) (Figure 5e). This technique leverages eEndoV to recognize and cleave at dI sites, creating a small nick in the DNA. Following this, *E. coli* DNA Polymerase I and DNA ligase are used to repair the nick while incorporating biotin‐14‐dATP into the DNA strand. The biotin‐labeled DNA fragments are then enriched using streptavidin beads for sequencing. This method is valuable for mapping dI in complex samples, such as mitochondrial DNA. It is worth noting that this method is inherently dependent on the double‐stranded structure of DNA and is therefore not applicable for detecting inosine modifications in RNA. In addition, as EndoV is a DNA repair enzyme, it may exhibit activity on certain types of DNA damage beyond dI, which could potentially compromise the specificity of dI‐seq in complex genomic samples.

Slic‐seq is a method for transcriptome‐wide identification of inosine‐containing RNAs, using a strategy similar in principle to that of hEndoV‐seq (Figure 5f). In Slic‐seq, RNA 3′ ends are oxidized by sodium periodate to block further reactions. EndoV then cleaves specifically at inosine sites, generating new 3′‐OH ends, which are then used for adapter ligation and sequencing (Wei et al. 2023). This makes possible precise detection of A‐to‐I RNA editing sites across the transcriptome. Slic‐seq and hEndoV‐seq are methodologically highly similar, both employing EndoV–mediated cleavage at inosine sites followed by adapter ligation to newly generated 3′‐OH termini. While their core strategies overlap, Slic‐seq incorporates procedural simplifications and has demonstrated enhanced sensitivity and base‐resolution mapping of inosine when compared to conventional RNA‐seq approaches. Accordingly, the choice between Slic‐seq and hEndoV‐seq may depend less on inherent differences in detection performance, and more on practical considerations such as protocol complexity, enzyme availability, and compatibility with downstream library preparation workflows.

In summary, the continuous development of inosine detection methods has resulted in both chemical and enzyme‐assisted approaches with distinct advantages and applications. Chemical reaction methods, which rely on molecular interactions, offer high reproducibility, reduced variability, and easy handling, making them particularly suitable for large‐scale or repetitive studies with minimal batch‐to‐batch variation. Additionally, chemical reagents are generally less expensive. The availability of ready‐to‐use derivatives further simplifies the process, reducing both time and technical costs, as no additional synthesis steps are required. In cases where proof of inosine presence is needed based on its chemical properties and inherent reactivity, chemical reactions provide a reliable and straightforward approach—sometimes the only viable option to directly demonstrate inosine presence through chemical principles.

By contrast, enzyme‐assisted methods offer high specificity and precise target recognition, making them especially effective for detecting low‐abundance RNA editing events and processing complex samples. Moreover, enzyme reactions typically occur under mild conditions, minimizing sample degradation and preserving RNA integrity. However, enzyme efficiency can be influenced by factors such as salt concentration and RNA structure, potentially leading to incomplete detection and diminished accuracy. Enzyme‐based methods also depend heavily on the activity and purity of the enzymes used, which can vary between batches, affecting the overall reproducibility of the results. Furthermore, adapter ligation or tailing procedures can further impact the consistency of the method. In general, enzymatic reactions are often less reproducible than chemical methods, especially when precision across multiple experiments is required. By contrast, chemical methods block reverse transcription by adducting compounds to inosine sites, resulting in shorter cDNA and loss of upstream sequence information. For experiments requiring upstream sequence data, enzyme‐assisted methods are more appropriate due to their ability to preserve full‐length cDNA.

### 3.3 Inosine Quantification Methods

**FIGURE 6:**
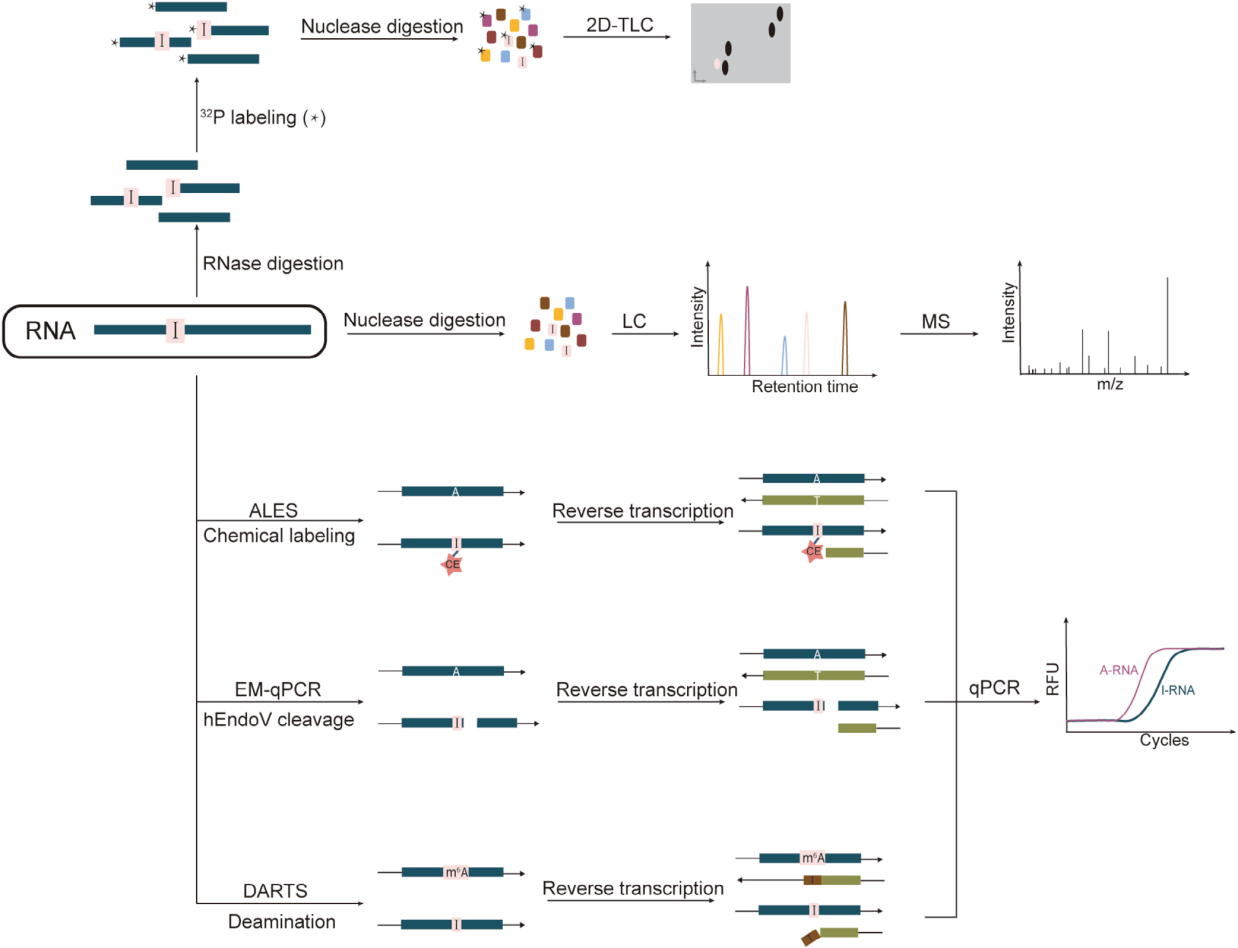
Schematic workflow of inosine quantification methods.

Based on the distinct chemical properties of inosine compared with other nucleotides, the main quantitative methods for detecting inosine modifications currently include two‐dimensional thin‐layer chromatography (2D‐TLC) and liquid chromatography‐mass spectrometry (LC‐MS; Figure 6). These methods can be used to quantify the abundance of inosine in specific RNA species. The 2D‐TLC method separates nucleotides based on their migration rates in a solvent to detect RNA modifications. RNA is first partially hydrolyzed by alkaline treatment and then labeled at the 5′ end with ^32^P. After labeling, RNA is fully digested into nucleotides by nuclease P1 (Bass 2024; Bass and Weintraub 1988; Kuchino et al. 1987; Paul 1998) and the nucleotides are identified by comparison with standards and quantified by measuring radioactivity. This approach is highly sensitive and requires only a small amount of RNA. It is also cost‐effective because expensive instrumentation is not required. However, it necessitates the use of radioactive labeling, with associated safety and disposal issues, and also requires extensive purification of the RNA sample prior to analysis. LC‐MS involves complete digestion of RNA into nucleosides, which are then ionized and further fragmented into specific ions by MS (Baquero‐Pérez et al. 2024; Feng et al. 2022; Tao et al. 2023). The retention time and mass‐to‐charge ratio (m/z) help determine the presence of inosine. Additionally, quantification of inosine can be achieved through an external calibration curve run in parallel. However, limitations of this technique include the requirement for HPLC and MS equipment, sensitivity issues arising when detecting inosine in low abundance in RNA species, and the need for extensive sample purification prior to analysis.

In addition to the methods mentioned above, some real‐time quantitative PCR (qPCR)‐based techniques have been developed for inosine quantification (Figure 6). One such method is acrylonitrile labeling‐mediated elongation stalling (ALES) (Ding et al. 2023), which builds on the ICE method discussed above. ALES selectively labels inosine, causing reverse transcription to stall at inosine sites, enabling quantification via qPCR. Another technique is endonuclease‐mediated qPCR (EM‐qPCR) (Tao et al. 2024). This method utilizes hEndoV to specifically cleave inosine‐containing RNA, causing reverse transcription to stall at inosine sites. The stalled products are then quantified by qPCR. Furthermore, the deamination‐assisted reverse transcription stalling (DARTS) method allows for the simultaneous detection of A‐to‐I editing and N6‐methyladenosine (m^6^A) modifications at the same RNA sites (Min et al. 2024). By leveraging the deamination activity of the TadA–TadA8e protein (an engineered version of the tRNA‐specific adenosine deaminase) and reverse transcription stalling at inosine, DARTS provides site‐specific quantification through qPCR, facilitating analysis of the interplay between these two modifications. These qPCR‐based methods can quantify the extent of editing at known inosine sites, making them valuable for targeted analyses, although they are not suited for broader genome‐wide investigations.

## 4 Conclusion

This is an exciting time to study A‐to‐I RNA editing because advances in detection techniques have opened up new avenues for research. The discovery of functionally significant A‐to‐I editing sites has illuminated their roles in regulating key biological processes such as neurotransmission, immune regulation, and tumorigenesis. Editing sites within coding regions can lead to protein recoding, while those in non‐coding RNA, including miRNAs and lncRNAs, are critical for controlling gene expression at multiple levels. These findings highlight the importance of RNA editing in maintaining cellular homeostasis and its potential involvement in disease pathogenesis.

Despite these insights, most editing sites remain functionally unexplored, making it essential to continue investigations into their broader biological roles. Understanding these roles relies on the ability to precisely detect and quantify A‐to‐I editing events. Therefore, the development of sensitive, accurate, and high‐throughput detection technologies is crucial for advancing research into RNA editing, and unlocking its full potential in both fundamental biology and medical applications.

It is important to acknowledge that no single method works best for all experimental conditions. Each detection method has its own strengths and weaknesses. Chemical‐assisted methods, while offering good specificity and simplicity, struggle with reactivity issues in complex nucleic acid samples. Notably, ICLAMP stands out as a promising solution. By using commercially available maleimide derivatives, which do not require complex synthesis, ICLAMP provides greater flexibility and efficiency for detecting inosine modifications, making it an improvement over other chemical methods.

On the other hand, enzyme‐assisted methods offer high specificity but are sensitive to RNA structure and experimental conditions, which limits their broader application. Early methods, such as those using RNase T1, required blocking guanosines, which made the process complicated. The introduction of EndoV improved specificity by removing the need for this masking step. However, enzyme‐assisted methods still face challenges due to the complexity of RNA structures, and because most improvements focus on the steps after enzyme cleavage rather than directly enhancing the enzymes themselves.

Importantly, chemically‐assisted methods focus primarily on modifying the chemical compounds used to assist in the detection of inosine for sequencing purposes. By contrast, enzyme‐assisted methods are typically developed to improve the processes involved in library preparation after enzyme cleavage because it is difficult to directly modify the enzymes themselves. This distinction highlights the key difference between these approaches: chemical methods evolve by enhancing the reactivity and specificity of the compounds, while enzyme methods refine the workflow to maximize efficiency and accuracy without altering the enzymes.

Although current RNA editing detection methods have not yet been implemented in clinical practice, their potential applications are substantial. We anticipate that sensitive inosine detection approaches could be used to quantify editing at disease‐associated sites, enabling the identification of diagnostic or prognostic biomarkers. In neurodegenerative diseases, where editing alterations may precede clinical symptoms, such methods may eventually facilitate early‐stage detection. In cancer research, tumor‐specific editing patterns could theoretically complement genomic profiling to support disease classification or therapeutic decision‐making. Furthermore, if site‐directed RNA editing strategies are developed for therapeutic purposes, inosine detection tools would likely play a key role in monitoring both on‐target and off‐target editing events. While these scenarios remain hypothetical, they underscore the importance of further developing inosine detection methods.

In summary, while current detection methods have expanded our understanding of A‐to‐I RNA editing, the next phase of research will require continued innovation in both technology and methodology. Future studies should focus on integrating these advancements with functional genomics and transcriptomics to elucidate the full spectrum of editing sites and their physiological relevance. Moreover, most existing strategies remain limited to endpoint measurements and are typically applied to bulk RNA populations, which may obscure cell type–specific editing heterogeneity and temporal dynamics. Emerging technologies—such as single‐cell RNA sequencing and real‐time editing reporters—represent promising directions to overcome these constraints. Nonetheless, their effective implementation in conjunction with existing inosine detection strategies requires addressing several technical challenges. For chemically assisted methods, key issues include nonspecific reactivity with endogenous RNA modifications such as pseudouridine, interference from intracellular proteins, and the incompatibility of high‐temperature or solvent‐dependent reactions with live‐cell environments. Enzyme‐based approaches, while generally more biocompatible, are constrained by limited catalytic stability, potential off‐target cleavage, and reduced accessibility to structured or low‐abundance RNAs within complex cellular contexts. Overcoming these barriers will be critical for extending RNA editing detection into spatially and temporally resolved biological systems.

## Author Contributions

**Yuxi Yang:** methodology (lead), visualization (lead), writing – original draft (lead), writing – review and editing (equal). **Masayuki Sakurai:** supervision (lead), writing – review and editing (equal).

## Conflicts of Interest

The authors declare no conflicts of interest.

## Related WIREs Articles

Substitutional A‐to‐I RNA editing

The role of RNA editing enzyme ADAR1 in human disease

Evolutionary driving forces of A‐to‐I editing in metazoans

Site‐selective versus promiscuous A‐to‐I editing

Adenosine‐to‐inosine RNA editing

## Data Availability

Data sharing is not applicable to this article as no new data were created or analyzed in this study.
